# Quantitative Live-Cell Imaging of Human Immunodeficiency Virus (HIV-1) Assembly

**DOI:** 10.3390/v4050777

**Published:** 2012-05-04

**Authors:** Viola Baumgärtel, Barbara Müller, Don C. Lamb

**Affiliations:** 1 Department of Chemistry, Center for NanoScience (CeNS) and Center for Integrated Protein Science, Munich (CIPSM), Ludwig-Maximilians-Universität München, Butenandtstr. 5-13, D-81377 München, Germany; Email: viola.baumgaertel@cup.uni-muenchen.de; 2 Department of Infectious Diseases, Virology, University Hospital Heidelberg, Im Neuenheimer Feld 324, D-69120 Heidelberg, Germany; 3 Department of Physics, University of Illinois at Urbana-Champaign, Urbana, IL 61820, USA

**Keywords:** HIV, assembly, fluorescence, microscopy, ESCRT, live-cell imaging

## Abstract

Advances in fluorescence methodologies make it possible to investigate biological systems in unprecedented detail. Over the last few years, quantitative live-cell imaging has increasingly been used to study the dynamic interactions of viruses with cells and is expected to become even more indispensable in the future. Here, we describe different fluorescence labeling strategies that have been used to label HIV-1 for live cell imaging and the fluorescence based methods used to visualize individual aspects of virus-cell interactions. This review presents an overview of experimental methods and recent experiments that have employed quantitative microscopy in order to elucidate the dynamics of late stages in the HIV-1 replication cycle. This includes cytosolic interactions of the main structural protein, Gag, with itself and the viral RNA genome, the recruitment of Gag and RNA to the plasma membrane, virion assembly at the membrane and the recruitment of cellular proteins involved in HIV-1 release to the nascent budding site.

## 1. Introduction

The late stages of HIV-1 replication comprise the assembly of virus particles from newly synthesized components at the plasma membrane of the virus producing cell and the release of progeny virus by a membrane fission event. Release is accompanied by maturation of the virion induced by cleavage of the main structural polyprotein Gag by the viral protease (PR) and is characterized by dramatic morphological alterations that render the particle infectious. These processes are orchestrated by the structural polyprotein Gag, which engages in numerous molecular interactions important for the process of virion formation. The polyprotein targets to the plasma membrane where it self-assembles into virus like particles (VLPs). Gag is further responsible for incorporating the viral RNA genome, envelope glycoproteins, viral enzymes and other virion components, as well as for recruiting cellular proteins essential for particle release. 

Structural and mechanistic aspects of HIV-1 particle formation have been studied for many years using various biochemical and virological approaches as well as electron microscopy (EM) and, more recently, electron tomography (ET). Taken together, these studies have yielded a wealth of information on virion composition and architecture [1], the localization of Gag-Gag interaction and assembly in various cell types and the functional role of Gag domains, nucleic acid, cellular factors or proteolytic processing events [2]. However, one important aspect still remains elusive, the kinetics of these highly dynamic processes. Electron micrographs display still images of various assembly and maturation stages, but cannot capture the continuity of events or visualize transient intermediates that may occur on a fast time scale. Furthermore, EM and ET are not well suited for the analysis of large numbers of individual particles or events in order to obtain statistically significant results on heterogeneous particle populations or on different events occurring in parallel. Biochemical studies are limited by the fact that virion assembly, release and maturation do not occur synchronously—neither between infected cells in a culture, nor between virions produced from an individual host cell. Attempts to artificially synchronize individual steps in order to investigate their dynamics by ensemble measurements have so far been unsuccessful.

More recently, fluorescent labeling of proteins and/or RNA in living cells in combination with sensitive fluorescence microscopic techniques have provided excellent methods for studying the dynamics of steps in the HIV-1 assembly process. Fluorescence microscopy is minimally invasive and the emitted fluorescence can be detected with high sensitivity, opening the way for various microscopy approaches. Application of these techniques has allowed the analysis of Gag trafficking and assembly in real time, providing insights into the time dependent intracellular localization of virus components, as well as the dynamics and order of events occurring at the viral budding site.

In this review, we will describe labeling strategies for HIV-1 and discuss various fluorescence techniques used for investigating virological processes. We will then focus on recent applications of quantitative fluorescence microscopy for elucidating dynamic steps in the assembly process of HIV-1.

## 2. Methods

### 2.1. Fluorescence Labeling Strategies for HIV-1

Observation of HIV-1 particle assembly using live-cell microscopy requires the attachment of a suitable label to a component of the virus particle. The fact that Gag is not only the main orchestrator of the HIV-1 assembly process, but also the most abundant component of the virion, renders it an attractive target for labeling approaches. The most widely used labeling strategy for live-cell applications, which has also been used to investigate HIV-1 assembly, is fusion to autofluorescent proteins (FPs). FPs comprise a color palette ranging from blue to the far red as well as photoactivatable and photoswitchable derivatives suitable for pulse-chase analyses or sub-diffraction fluorescence microscopy [3,4,5,6]. In contrast to chemical labeling with synthetic dyes, employment of genetically encoded FPs results in attachment of the label at a well-defined position and avoids staining steps that may be difficult to control. Hence, the effect of the label on the functionality of the virus is reproducible and can be characterized. However, one should be aware that, depending on the fusion context, a significant fraction of FPs may be non-fluorescent due to misfolding of the protein, immature chromophores, or chromophores in a dark state. Since approximately 2400 Gag molecules are incorporated into a single HIV-1 particle [7,8], labeling with FPs still permits sensitive detection of individual assembly sites even when spiking the virions with non-fluorescently labeled Gag molecules. Codon-optimized versions of HIV-1 Gag, which can be overexpressed in the absence of other HIV-1 proteins and are tagged at the C-terminus by fusion to FPs, have been described [9]. FPs have also been inserted between the N-terminal matrix (MA) domain and capsid (CA) domain of the Gag polyprotein, allowing labeling of Gag in the context of the complete viral genome [10,11]. While FP labeled virus derivatives have been instrumental in studying kinetics of Gag assembly at the plasma membrane (see e.g., Section 3.2), one of the disadvantages of FPs is their size of ~27 kDa, resulting in reduced replication efficiency of the modified virus under most conditions. The requirement for chemical maturation of the fluorophore also limits detection of the earliest steps in trafficking of newly synthesized proteins. An alternative labeling approach is the small tetracysteine-tag (TC-tag), which comprises only 6–12 amino acids and can be stained using biarsenical dyes [12]. A drawback of this method is the limited selection of cognate dyes currently available and the requirement for an additional chemical labeling step, which can result in background staining. More specific labeling is usually achieved with larger protein based tags that can react intracellularly with an added fluorescently labeled ligand. An example of this approach is the SNAP-Tag, a 20 kDa variant of the human O6-alkylguanosyltransferase (hAGT), which undergoes covalent self-labeling with various benzylguanine (BG) derivatives [13]. Both TC-tag [12] and SNAP-tag [13,14] have been employed to generate labeled Gag derivatives, expressed either alone or in the viral context, but use of these derivatives in live-cell imaging analyses has so far been limited. The large variety of FPs with different spectral properties makes it possible to perform multi-color experiments, which allow a combined detection of labeled Gag with other fluorescently-labeled virion components or cellular factors involved in the assembly process. Surrogates of the HIV-1 genomic RNA can be labeled for live-cell imaging by including sequence motifs specifically recognized by RNA binding proteins such as the bacteriophage MS2 coat protein. FP-tagged versions of the RNA binding protein attach to the cognate RNA motif, thereby labeling the RNA in live cells [15,16]. This approach opened the possibility for quantitative analyses of Gag-RNA interactions by fluorescence imaging [17].

### 2.2. Fluorescence Microscopy and Spectroscopy Methods

**Figure 1:**
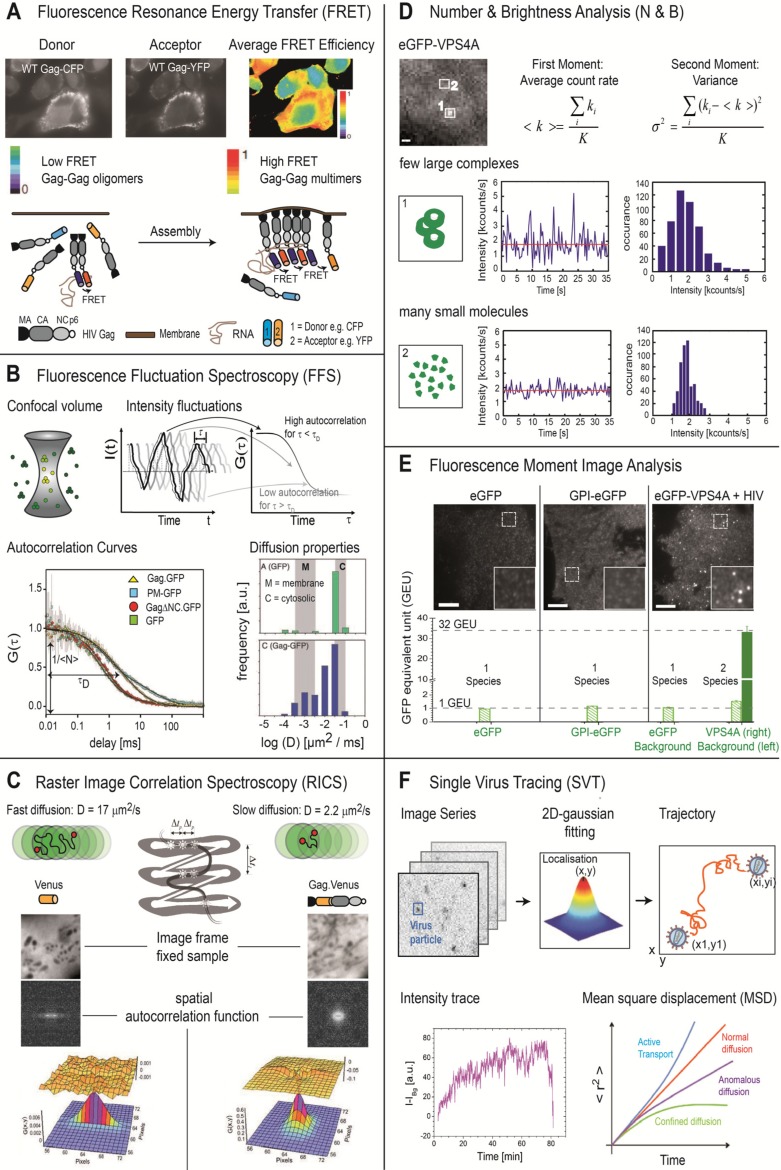
Selective methods for quantitative fluorescence analysis of HIV-1 assembly. Numerous fluorescence methods are available for investigating the dynamics of viral processes and virus-cell interactions. A few of them are highlighted in the figure. (**A**) FRET can be used to investigate the spatial distribution of molecular interactions in live cells. Using Gag.CFP and Gag.YFP, Hogue *et al.* investigated the interaction of WT and mutant Gag molecules in the cytosol and plasma membrane using FRET [18]. (**B**) FFS uses fluctuations in fluorescence intensity to determine the mobility of fluorescently labeled molecules. From FFS experiments on GFP-tagged Gag with deletion of the nucleocapsid (NC) domain, plasma membrane bound GFP, and cytosolic GFP, Larson *et al.* demonstrated that the mobility of Gag is significantly decreased in the plasma membrane as shown by the shift in the correlation curve to longer times. Thus, with FFS, they could investigate the role of NC in the assembly process [19]. (**C**) RICS measures the mobility of fluorescence molecules by utilizing the correlations between pixels in a raster‑scanned image. The RICS autocorrelation function for Venus (left) and Venus‑labeled Gag (right) are shown. (**D**) N&B Analysis determines the number and molecular brightness of the labeled biomolecules from the fluctuations in fluorescence intensity over time. A small number of large bright complexes will show a larger variance in the measured fluorescence intensity than a larger number of dim molecules, even though the average fluorescence intensity may be similar. (**E**) Fluorescence moment image analysis uses the fluorescence intensity distribution of an image to determine the number and brightness of complexes in the image. A higher-order moment analysis was performed by Baumgärtel *et al.* to estimate the number of VPS4 molecules that interact with nascent HIV-1 assembly sites [20]. (**F**) SVT can be used to following individual viruses as they enter or exit living cells. The position of the virus is determined in each frame by fitting the point-spread function to a 2D Gaussian, yielding the trajectory. From the trajectory information, the fluorescence intensity during the trajectory or the diffusional behavior can be determined [21].

Once viral and/or cellular components have been fluorescently labeled, a number of fluorescence microscopy approaches can be used to investigate the dynamics of protein complexes or single viruses during assembly. Image based techniques like Total-internal Reflection Fluorescence (TIRF) microscopy, Spinning-disc Confocal Microscopy (SDCM) or super-resolution fluorescence microscopy exploit the spatial distribution of the fluorescence intensity to extract the position and/or brightness of a fluorescence object. In contrast, Förster Resonance Energy Transfer (FRET) uses the spectral or fluorescence lifetime information at a single position or per pixel to gain information on the conformation or interaction of biomolecules. Fluorescence fluctuation spectroscopy (FFS) methods analyze the temporal changes of the fluorescence intensity to gather information over the number of particles in the observation volume, their brightness and mobility. Interactions between proteins can be investigated using cross-correlation methods. Within this classification, Raster Image Correlation Spectroscopy (RICS) plays a special role as it analyzes both the spatial and temporal signal fluctuations within a raster scanned image, combining the strengths of FFS methods with imaging information. An overview of the various methods discussed in this review is given in (Figure 1) and (Table 1).

**Table 1:** Potential applications of quantitative fluorescence imaging techniques.

Method	Potential Applications
Fluorescence Resonance Energy Transfer (Figure 1A) [22]	Spatial and temporal investigations of molecular interactions (Gag-Gag interactions, membrane microdomain clustering [18,19,23]
	Conformational dynamics
Fluorescence Fluctuation Spectroscopy (Figure 1B) [24,25,26]	Binding kinetics and affinities
	Determination of the mobility of molecular complexes (Gag-Gag interactions in the cytosol [19])
	Detection of molecular interactions
	Mapping of the local environment of the cell through its influence on the diffusion properties
	Determination of fluorophores per complex (Gag stoichiometry in VLPs [27])
Raster Image Correlation Spectroscopy (Figure 1C) [28,29,30]	Binding kinetics and affinities
	Determination of the mobility of molecular complexes
	Detection of molecular interactions
Number and Brightness Analysis (Figure 1D) [31]	Determination of molecular or complex concentration, brightness and stoichiometry at each pixel of an image from an image series [27]
Fluorescence Moment Image Analysis, Image Correlation Spectroscopy (Figure 1E) [32,33]	Determination of molecular or complex concentration, brightness and stoichiometry and presence of different subpopulations from the intensity distribution over a single image (cytoplasmic Gag-Gag interactions [34])
Live cell imaging combined with Single Virus Tracing (Figure 1F) [35]	Analysis of entry and release pathways [36]
	Kinetics of virus entry and assembly [20,37,38]
	Dynamics of intracellular trafficking of virus or viral proteins
	Interaction of viral proteins with host factors [20,39].

#### 2.2.1. Wide-Field and TIRF Microscopy

The rapid time scale of the processes involved in virus-cell interactions makes high time resolution essential. Wide-field microscopy is the most straightforward and fastest imaging method available and has been used to track viral particles, for instance, as they enter living cells [40,41]. Wide-field microscopy can be performed with very high sensitivity as high numerical objectives can be used to efficiently collect the emitted fluorescence, back-illuminated EMCCD cameras with the highest available detection quantum-yield can be used for sensitive detection and very few optical elements are necessary in between. However, for measuring processes at the plasma membrane using fluorescently labeled proteins that exist mostly in the cytoplasm, the background fluorescence resulting from the large amount of newly expressed protein in the cytoplasm is too high.

In contrast, TIRF microscopy is well suited for the observation of events that take place at the plasma membrane [42,43], such as HIV-1 assembly [37,38]. In TIRF microscopy, the excitation light reaches the interface between the coverslip and the cell at an oblique angle to the surface resulting in total reflection of the excitation beam accompanied by the formation of an evanescent wave at the interface. The intensity of this wave decays exponentially with increasing distance from the interface, resulting in excitation of fluorophores only within a distance of ~100–200 nm from the coverslip. Therefore TIRF microscopy visualizes mainly molecules at the plasma membrane with very low background from the cytoplasm, as only a small region of the cytoplasm is excited by the evanescent wave. Similar to wide-field microscopy, TIRF microscopy can be performed with very sensitive detection.

However, when using the fluorescence intensity measured in TIRF microscopy to infer information regarding the number of detected molecules, caution has to be taken. The steep exponential decay of the intensity of the evanescent field renders the method very sensitive to the axial position of the fluorescent molecules. Fluctuations in the fluorescence intensity due to movement of the plasma membrane can cause difficulties in data interpretation when fluorescence intensity is used as a quantitative measure for assembly of complexes at the plasma membrane. To address this problem, TIRF microscopy can be combined with wide-field observation [38,44]. Even when using high numerical objectives, wide-field microscopy has a depth of focus on the order of 1 μm or more, significantly exceeding the penetration depth of the evanescent wave in TIRF microscopy. By comparing fluorescent intensities in TIRF microscopy and wide-field microscopy, it can be discriminated whether an observed intensity increase arises from new molecules arriving at the assembly site or from axial-movement of the site. Given sufficient signal-to-noise ratio in the wide‑field channel, one can also use the ratio of fluorescence intensities in wide-field and TIRF microscopy to extract the axial position of the particle [44]. Calibration is required for determining the absolute axial position of the particle above the coverslip, but this step is dispensable if only changes in the *z*-position are of interest.

#### 2.2.2. Spinning Disc Confocal Microscopy

The main disadvantage of TIRF microscopy is that it can only detect events occurring at or near the coverslip. Thus, other microscopic techniques need to be applied to investigate processes that occur within the cytoplasm or at the dorsal plasma membrane. Confocal microscopy is the method of choice as it provides sufficient contrast between the fluorescence signal of interest and the background fluorescence while allowing high time resolution to measure the dynamics of transient processes in HIV-1 assembly. Typically, either a fast confocal laser scanning microscope (CLSM) or a spinning disc confocal microscope (SDCM) is used. When the process to be observed is slow enough to allow collection of multiple successive *z*-planes, three-dimensional information can also be extracted from the *z*-stack data [45,46,47]. For example, the assembly of HIV-1 at the dorsal plasma membrane [38] and the assembly of Murine Leukemia Virus in the direction of cell-cell contacts [48] were measured using SDCM in combination with very sensitive EMCCD cameras.

#### 2.2.3. High Resolution Fluorescence Microscopy

During the last few years, advances in super-resolution fluorescence microscopy made it possible to collect optical images with spatial resolution beyond the diffraction limit. This represents a very promising tool for investigating viruses and subviral structures, as their dimensions are smaller than the diffraction limit of light microscopy. Two classes of super-resolution methods have been described: methods where the detection volume of the measured fluorescence is smaller than the diffraction limit, as in STimulated Emision Depletion (STED) [49] and REversible Saturable OpticaL Fluorescence Transitions (RESOLFT) [50] and methods that use the localization of individual fluorophores to reconstruct super-resolution images such as Stochastic Optical Reconstruction Microscopy (STORM) [51] and Photo-Activated Localization Microscopy (PALM) [52,53]. Using FP‑tagged rotavirus capsid protein assemblies, Willig *et al.* have demonstrated that STED can be used to visualize individual 70 nm sized VLPs [54]. PALM or STORM has been used to visualize individual HIV Gag assembly sites [14,52]. Manley and coworkers combined PALM and single‑particle tracking to investigate the motion of HIV Gag and the vesicular stomatitis virus glycoprotein (VSV-G) labeled with EosFP at the plasma membrane in live cells [55]. Lehmann *et al.* used multi-color super-resolution imaging to visualize the interaction of the host cell restriction factor tetherin with HIV-1 assembly sites. They observed clusters of tetherin forming close to viral budding sites and analyzed the amount of tetherin molecules involved and the requirements for cluster formation [56]. Until now, super-resolution experiments have mainly been performed on fixed samples. However, by using a photoswitchable protein that can be cycled thousands of times, super-resolution microscopy can now be performed in live cells using low illumination powers [57]. The application of super-resolution microscopy methods in virology is currently in its infancy but promises to be an essential tool for investigating viral processes in live-cells in the near future.

#### 2.2.4. Förster Resonance Energy Transfer

The process of HIV assembly involves numerous interactions of virion components with each other as well as with factors from the host cell. Förster Resonance Energy Transfer (FRET) is an excellent tool to study molecular interactions in live cells [22]. FRET involves the radiation-less transfer of energy between a suitable pair of chromophores (donor-acceptor) due to dipole-dipole interactions [58,59]. The method is sensitive to the separation of the two chromophores in the range of 2–10 nm and is thus often used to investigate molecular processes and interactions, being referred to as a molecular ruler [60]. Detection of FRET can be performed by monitoring the decrease in donor intensity or donor fluorescence lifetime in the presence of an acceptor molecule, or the enhanced fluorescence intensity of the acceptor in the presence of a donor molecule. FRET Experiments can be done in solution, at a point and also in imaging mode. By attaching an appropriate pair of FPs, FRET can be used to analyze protein interactions in living cells and thus represents a useful tool for studying the interactions between viral and cellular proteins as well as oligomerization and multimerization events in the viral assembly process. For example, it has been employed to investigate Gag-Gag interactions in the retroviral assembly pathway [10,19,37,61] (Figure 1A). Recently, FRET has also been used to monitor proteolytic maturation within HIV-1 particles during cell-to-cell transmission. The change in FRET signal during maturation of the nascent HIV-1 particles was then used to verify the hypothesis that immature particles can be taken up via cell-to-cell contacts and maturation can take place in intracellular compartments of the CD4^+^ acceptor T cell [62].

#### 2.2.5. Fluorescence Fluctuation Spectroscopy

Another group of fluorescence methods that are very powerful for investigating mobility, particle concentration, molecular brightness and the interaction of molecules is Fluorescence Fluctuation Spectroscopy (FFS) [63,64,65]. FFS analyzes the non-stochastic fluctuations in the fluorescence intensity arising, for example, from thermodynamic fluctuations in the number of molecules in the focus of the microscope. The fluctuations are analyzed using an autocorrelation analysis. From the resulting autocorrelation function, the diffusion coefficient and average number of molecules in the volume can be determined (Figure 1B). A number of FFS methods have been developed in the last decades that analyze either temporal and/or spatial fluctuations in fluorescence signals. Many FFS methods analyze the fluorescence data collected from a single point. In live-cell imaging, two powerful, recently developed FFS methods can be used namely Raster Image Correlation Spectroscopy (RICS) [28,29,66] and Number and Brightness analysis (N&B) [31]. 

RICS utilizes the fixed relationship between the 2D separation of pixels in a raster-scanned image and the time delay between data collection at the respective pixels (Figure 1C). A scanner with good linearity is required for measurement of the spatiotemporal correlations. Although the RICS autocorrelation function is determined from a single scanned image, typically 50–100 images are collected from the same region and averaged to provide good statistics. The shape of the RICS autocorrelation function carries information regarding the mobility of the diffusing molecules captured in the image. Compared to Fluorescence Correlation Spectroscopy, RICS has the advantage that it is a scanning technique and hence, the excitation power is distributed within the cell, minimizing photobleaching. The N&B analysis can be performed on the same images but is based on the intensity fluctuations undergone by individual pixels over the course of the movie, which carry information about the number and brightness of the molecules. N&B assumes a single diffusing species and extracts an effective molecular brightness and number of molecules from the average value and variance of the fluorescence signal (Figure 1D). By comparing N&B data obtained for an FP fusion protein of interest to measurements on FP alone, it is possible to estimate the average size of oligomers present in a cell. One advantage of the RICS and N&B analyses is that they are performed on images and thus the data also contain image information that can be used for other purposes. Whereas RICS utilizes the precise relationship between timing and spatial position of a raster-scanned image, N&B can be performed on any imaging data (e.g., from SDCM or wide-field setup with a camera for detection) provided the integration time is fast enough to detect the fluctuations.

FFS methods are based on the temporal fluctuations in fluorescence intensity, whereas Image correlation spectroscopy (ICS) is based on spatial fluctuations within an image. The fluorescence intensities measured at different positions of an image display a distribution of fluorescence intensities due to the varying density of fluorophores at these positions. By analyzing these spatial fluctuations, the average number of particles and molecular brightness of fluorophores in the image can be calculated [67,68]. A number of image correlation methods has been developed to extract information from images (for a review see [32]). A method recently used to investigate the interaction of nascent HIV-1 assembly sites with the cellular protein VPS4 is fluorescence moment image analysis [20]. This method extracts information over the number of species, the number of molecules of each type and the molecular brightness of the different species from the moments of the fluorescence intensity distribution of an image. When only one fluorescently labeled species is present, the average (1^st^ moment) and variance (2^nd^ moment) are sufficient to describe the fluorescence intensity distribution. When two species are present, the higher-order moments, Skewness (3^rd^ moment) and Kurtosis (4^th^ moment), are required to extract the numbers and molecular brightness of the two co‑existing species (Figure 1E) [33].

### 2.3. Single Virus Tracing

After performing time resolved imaging in two or three spatial dimensions, a wealth of information can be extracted using different analysis methods. Given that individual virions or subviral particles can be resolved, single virus tracing (SVT) is an invaluable tool for following the dynamics of the observed processes (Figure 1F). There are two basic approaches for tracking single particles: one is to track a particle in real-time using a feedback loop [69], while the second, currently preferred method involves tracking *ex post facto* [20] from image sequences, either manually or using automated tracking software. Tracking involves first locating the individual particles in each frame and channel collected during a movie. If the particles are smaller than the point-spread-function of the microscope, this is often done with the aid of a spot enhancing filter and the use of a Kalman filter (e.g., [45,70,71,72,73]). The main difficulty in SVT is the correspondence finding, which maps the movement of each particle between consecutive frames. This is challenging since new particles can assemble or appear, signals from two particles can merge or split, or particles can disappear from the observation plane. Hence, the verification of trajectories by visual inspection is imperative. Based on the coordinates and intensities determined for each particle for all consecutive time points, its intracellular localization, co‑localization with cellular organelles or structures as well as changes in fluorescence intensities indicative of dissociation or association processes can be analyzed. In addition, the 2D or 3D instantaneous velocity of the particle and the type of motional behavior (diffusion, corralled diffusion or transport) can be determined by a mean-squared-displacement analysis [21,74,75,76] providing detailed insight into the mechanisms and dynamics of cell-virus interactions.

## 3. Applications of Quantitative Fluorescence Microscopy in Elucidating HIV-1 Assembly

### 3.1. Initiation of the Assembly Process

**Figure 2:**
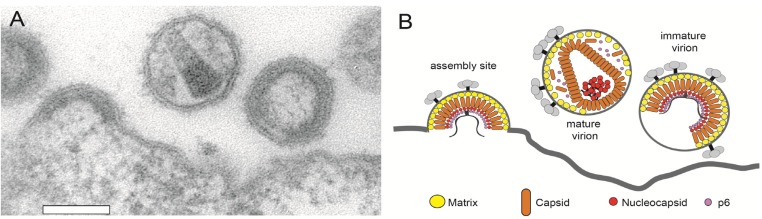
Architecture of the HIV-1 budding site and of released particles. (**A**) Electron micrograph of a HIV-1 budding site showing immature and mature virions at the plasma membrane of a virus producing T-cell. Scale bar 100 nm. (**B**) Schematic representation of the structures shown in (A).

The HIV-1 Gag polyprotein, consisting of four functional domains, is the only viral protein required for assembly and budding of VLPs from the plasma membrane of virus producing cells. The N‑terminal MA domain is responsible for membrane targeting and membrane binding, while the CA domain provides protein-protein interactions essential for formation of the immature Gag shell and the mature conical capsid. The nucleocapsid (NC) domain of the HIV-1 Gag polyprotein is essential for viral RNA binding [77], which in turn plays an important role in the assembly process by concentrating Gag molecules and promoting conformational changes relevant for membrane association. The C-terminal p6 domain recruits the cellular ESCRT machinery required for virus release. During HIV-1 assembly, Gag polyproteins are transported to the plasma membrane and assemble into an outward curved structure, which grows into a spherical membrane enveloped bud that finally pinches off from the cell membrane (Figure 2). HIV-1 particles do not contain a precisely defined number of Gag molecules and HIV-1 preparations show a rather broad distribution of virion diameters. Electron tomography analyses have revealed that the protein shell of immature HI virions is an incomplete sphere. Based on these results, it was estimated that a virion comprises an average number of ~2400 Gag molecules [7,8]. Quantitative measurements of VLPs using fluorescence brightness analysis showed that the number of Gag molecules within one VLP varied with the expression level of Gag in the host cells from 750 to 2500, while the diameter of VLPs did not change, also indicating varying degrees of shell completeness [27].

The retroviral assembly process starts with the oligomerization of Gag. In the HIV-1 replication cycle, this step is coupled to the recognition of dimeric viral RNA genome, which is recruited by Gag to the viral assembly site [78,79]. With the aid of two-photon FRET, Larson *et al.* [19] demonstrated the oligomerization of Rous Sarcoma Virus (RSV) Gag not only at the plasma membrane but also within the cytosol. Initiation of HIV-1 assembly at the plasma membrane is apparently governed by an intricate relationship between Gag-Gag, Gag-RNA and Gag-lipid interactions. Analyzing cytosolic and membrane diffusion of Gag.GFP by FCS confirmed the formation of cytosolic Gag-Gag complexes and allowed analysis of the role of the NC domain and the membrane-binding domain of MA within this process [19]. The interaction of Gag with RNA appears to be an important step in the initiation of the assembly process with RNA acting as a scaffold for the binding of Gag molecules [77]. Gag-RNA interactions also appear to convert Gag from a compact conformation into an extended structure required for virus assembly [80]. However, Gag-RNA complexes formed in the cytosol contain only a low number of Gag molecules. Co-immunoprecipitation assays verified that Gag predominantly forms low order oligomers in complex with viral RNA in the cytosol whereas higher order Gag complexes are only detected at the host cell membrane and are dependent on the presence of a membrane interaction domain. In a recent study, fluorescence brightness analysis based on two-photon FFS [34] revealed that the oligomerization of cytosolic Gag is concentration dependent and leads to large cytosolic complexes when myristoylation of Gag is prevented by mutagenesis of the accepting glycine residue.

### 3.2. HIV-1 Assembly at the Plasma Membrane

**Figure 3:**
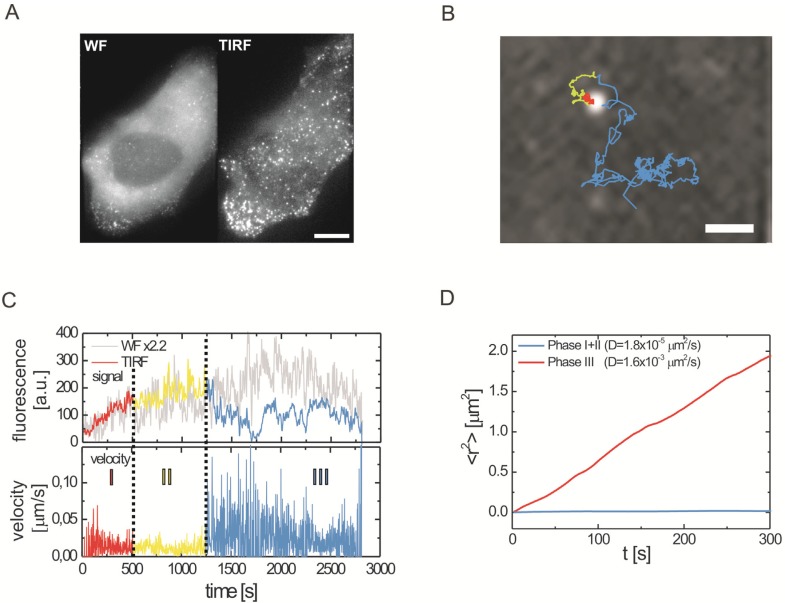
Imaging Gag assembly at the plasma membrane by wide-field (WF) and TIRF microscopy.(**A**) A HeLa cell expressing HIV.eGFP was imaged in WF (left) and TIRF (right) mode 25 hours post transfection; Scale bar, 5 µm (**B**) 2D trajectory of one single HIV-1 particle during assembly color coded according to the three different assembly phases shown in C; Scale bar, 1 µm. (**C**) Average background corrected WF (grey) and TIRF (colored) intensity traces (top) and instantaneous velocity plot (bottom) illustrating the three phases of HIV-1 assembly: phase I (red), a rapid increase in intensity and nearly no movement; phase II (yellow), an intensity plateau with slow movement; phase III (blue), a decrease in intensity correlated with a strong increase in movement or disappearance of the viral particle. (**D**) Mean square displacement (MSD) plot clearly showing the change in motional behavior from phase I/II to phase III, defining the release time point. All images adopted from Ivanchenko *et al.* [38].

The combination of fluorescently labeled virus derivatives with sensitive TIRF microscopy allowed the quantitative analysis of HIV-1 budding site formation at the plasma membrane of HeLa cells. Gag expressing cells display a diffuse cytoplasmatic staining followed by the appearance of fluorescent punctae at the plasma membrane that accumulate over a period of ~2 h (Figure 3A). The maximum intensity of the fluorescent punctae is comparable to that of individual fluorescent virions indicating that punctae represent individual assembly sites. By following the fluorescence intensity over time for many individual assembly sites, distinct phases during the assembly process could be distinguished (Figure 3B,C). At the majority of budding sites, the fluorescence intensity initially follows a saturating exponential, indicating gradual recruitment of Gag molecules. Delivery of larger pre-assembled structures to the membrane was not observed while the existence of smaller pre-assembled structures (consisting of 5% of the total labeled Gag signal or less) cannot be ruled out. Quantitative evaluation of 309 traces revealed an average rate constant of 0.0043 s^−1^ for this initial phase when Gag.GFP is expressed in the viral context resulting in 90 % completion within ~9 min. An average time of 7 min was determined for the assembly of Gag.GFP expressed alone [37]. Whether this small difference is due to a difference in expression context or other experimental conditions remains to be determined. Assembly rates remained unaltered when virion release was inhibited by mutation of the late domain motif or upon blocking virus maturation by introduction of a mutation in the PR active site [38]. Live‑cell TIRF imaging of Gag together with labeled viral RNA revealed that the nucleation event of assembly site formation involves a Gag dependent immobilization of viral genomes at the plasma membrane. Initial RNA recruitment apparently occurs through a small number of Gag molecules (*i.e.*, below the detection limit of the live-cell analyses performed) and the immobilized complex then serves as a nucleation site for the recruitment of further Gag molecules [17]. To investigate whether Gag arriving at the nascent budding sites was derived from the cytosolic pool or from plasma membrane associated Gag molecules, experiments were performed employing the photoconvertible FP mEOS. Using a wavelength of 405 nm for TIRF excitation, plasma membrane associated Gag.mEOS was photoconverted from green to red fluorescence. Analysis of budding sites formed shortly after photoconversion showed that Gag recruitment to the assembly site occurred mainly from the cytosolic pool rather than by lateral membrane-diffusion of Gag molecules binding to the surrounding membrane [38]. At the end of the exponential assembly phase, fluorescence intensity reaches a plateau. Photobleaching experiments revealed that Gag molecules are no longer recruited to the assembly site during this phase [37]. Characterization of viral release is hampered by a limitation inherent to TIRF microscopy, namely, that only the plasma membrane facing the coverslip can be visualized. Thus, many newly formed virions in the observation plane become trapped between membrane and glass surface and the budding process is not easily detectable.

Jouvenet *et al.* induced acidification of the cytosol by raising the external CO_2_ pressure and used the pH sensitivity of pHluorin-tagged Gag to show that the majority of virions were disconnected from the cytosol within approximately 30 min [37]. Unfortunately, this approach does not allow visualization of actual budding events in real time. A different approach was taken by Ivanchenko *et al.* [38], who inferred HIV-1 release through a change in particle motility or by the sudden disappearance of the particle. This has the advantage that it can be performed with high temporal resolution; however, fission events are not directly visualized and care needs to be taken to distinguish release from endocytotic uptake. Release of individual particles can be verified by a mean-squared displacement analysis, provided the virus could be tracked for several frames after release (Figure 3D). For the particle analyzed in Figure 3D, random Brownian motion (without active transport) was observed with a diffusion coefficient of 1.6 × 10^−3^ μm^2^/s, a motion that is much too fast to describe free diffusion of a particle the size of HIV-1 in the cytoplasm. Based on these experiments, release was inferred to occur with an average delay of ~15 min after the completion of Gag assembly, suggesting that additional processes may take place during the plateau phase [38].

### 3.3. ESCRT Recruitment and HIV-1 Release

HIV-1 particles are released by abscission of the enveloped virus bud from the plasma membrane of the virus-producing cell. This process is mediated by proteins that are part of the cellular endosomal complex required for transport (ESCRT) machinery, which are recruited to the viral budding site through interactions with Gag. ESCRT is a system of interacting protein complexes involved in various membrane fission events, most notably in the abscission of intralumenal vesicles forming a multivesicular body (MVB) and in cytokinesis [2,81,82,83]. While, in multivesicular budding, ESCRT-0 is responsible for the recruitment of monoubiquitinylated cargo proteins, ESCRT-I and -II apparently cooperate in a further translocation of the cargo to the vesiculation site and in the initial membrane bending. ESCRT-III proteins assemble into membrane-associated multimeric structures in the area of the bud neck and mediate membrane deformation, which promotes the fission event [84,85]. The AAA-ATPase VPS4 is required in the final stages of the budding process where it catalyzes dissociation of membrane bound ESCRT-III assemblies, thereby recycling the individual subunits for further use [86,87]. It has been well established that HIV-1 Gag can interact with the ESCRT components TSG101 and ALIX through ‘late domain’ motifs within p6 and that the virus exploits ESCRT to mediate bud abscission. However, parts of the machinery, in particular ESCRT-0, -II and some components of the ESCRT-III complex, have been shown to be dispensable for HIV-1 release [88,89]. While the assembly of a spherical Gag shell and the abscission of the membrane neck between virus and cell were originally assumed to be consecutive processes, the observation that the Gag shells of HI virions only line approximately 2/3 of the lipid envelope whereas the Gag shell is more complete in late-domain deficient mutants [7] suggested that Gag assembly and function of the ESCRT factors involved may be cooperative forces that mediate the membrane fission process. Recent live-cell imaging analyses of the recruitment of ESCRT components and ESCRT associated factors to the budding site have yielded information on the order of events occurring at retroviral budding sites [20,39].

**Figure 4:**
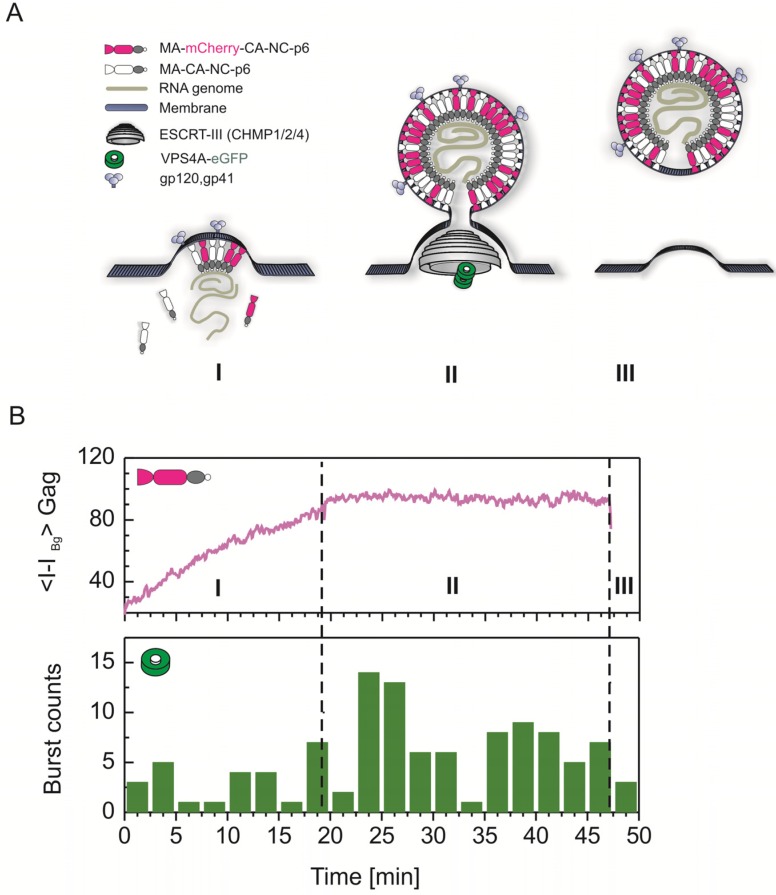
Interaction of VPS4 with HIV-1 assembly sites. (**A**) Model illustrating the interaction of the AAA-ATPase VPS4 during the three different HIV-1 assembly phases, based on several independent experimental studies including recent live-cell imaging results from Baumgärtel *et al.* [20] and Jouvenet *et al.* [39]. (**B**) Average kinetic scheme for Gag.mCherry assembly derived from experimental data (top). Histogram of time points during which VPS4A-Gag interactions and ATPase activity could be monitored at the membrane (bottom). The majority of VPS4A activity took place during phase II of the assembly process before the actual release of the virus [20]. Figure adapted from Baumgärtel *et al.* [92].

These experiments are challenging, since fusion to FPs renders most ESCRT proteins dysfunctional or dominant negative (dn). Thus, their expression levels and functionality have to be carefully controlled in each case. By employing stable cell lines expressing low levels of individual FP-tagged ESCRT proteins, Jouvenet *et al.* showed that the ESCRT associated protein ALIX accumulated simultaneously with Gag at retroviral assembly sites [39]. In contrast, ESCRT-III proteins as well as VPS4 were only transiently recruited to the budding site after the accumulation of Gag molecules had ceased (Figure 4).

Based on *in vitro* reconstitution experiments using purified yeast ESCRT proteins and model membranes, it had been proposed that membrane abscission is mediated by the concerted action of ESCRT-I, -II -III assembling in the neck of the nascent vesicle, while VPS4 is only required to catalyze recycling of ESCRT-III complexes [90,91]. In contrast, data from live-cell imaging studies suggest a more active role of VPS4, at least in the case of HIV-1 release. First, ESCRT-III is still recruited to the viral budding site in the presence of dnVPS4 while failing to mediate HIV-1 release under these conditions [39]. Secondly, a detailed analysis of the time points of VPS4 recruitment indicated transient recruitment of VPS4 after completion of Gag assembly but before virus release is detected [20]. Both findings are compatible with a model in which VPS4 contributes to the formation or the transient stability of an ESCRT-III assembly required for membrane abscission (Figure 4A). This is also in agreement with the observation of electron dense structures, possibly representing non‑functional ESCRT assemblies, in the neck region of late HIV-1 buds by electron microscopy upon knock-down of the ESCRT-III component Chmp2 required for VPS4 recruitment [89].

Viral bud neck constriction involves a large conformational reshaping of the plasma membrane, leading to an energetically unfavorable curvature prior to the final release of the virus. Thus, it can be considered as an energy consuming process and the energy involved was modeled in terms of neck diameter, radius of curvature and protein contributions by Fabrikant *et al.* [84]. While Gag in the absence of ESCRT is capable of assembling into spherical buds arrested before the membrane neck fission event, it is still unclear to what degree the growing Gag lattice, membrane bound assemblies of ESCRT proteins and the specific lipid composition of the membrane at retroviral budding sites cooperate to achieve membrane remodeling, curvature induction and fission during the regular HIV-1 budding event [23,93,94]. Further significant advancements can be expected from the development of improved labeling strategies allowing sensitive detection of fully functional proteins of interest and in particular from the development of methods yielding a real-time microscopic readout of membrane fission.

## 4. Future Directions

The application of quantitative live-cell imaging to investigate virological processes is in its infancy. Several recent developments in microscopy can be expected to have a profound impact on virological research. With improvements in reliability and sensitivity, SDCM will be suitable to investigate rapid processes in the life cycle of viruses that occur within the cytoplasm or at the medium exposed surface of the cell. A recent breakthrough in imaging is super-resolution microscopy. As most viruses and viral components are smaller than the diffraction limit of optical microscopy, super‑resolution methods open new opportunities to observe viral structures and interactions previously not accessible to optical microscopy. There is also a large effort to combine fluorescence and electron microscopy techniques in order to obtain correlative images that capture in detail both dynamic and structural aspects of biological processes. Using a correlative approach, Larson *et al.* [95] have demonstrated the correlation of fluorescently labeled punctae at the plasma membrane of HIV-1 Gag expressing cells with bulging budding structures detected by SEM of fixed samples. More recently, individual HIV-1 particles attached to a host cell [96] or undergoing cytoplasmatic entry [97] have been localized in electron tomograms based on correlative fluorescence microscopy. While the fast time scale of events in HIV-1 budding site formation and ESCRT recruitment still presents a challenge for such approaches, it can be envisioned that these emerging techniques will significantly contribute in the following years to a full understanding of the relation between molecular architecture and function for structures involved in HIV particle formation. Another promising microscopy method for investigating viral assembly in live cells is light-sheet fluorescence microscopy [98,99,100]. Here, a second objective is used to excite the sample in a direction perpendicular to the axis of observation. Hence, only a thin sheet in the focus of the microscope is illuminated and there is no out-of-focus photobleaching. Highly sensitive wide-field detection can also be used throughout the cell as the axial resolution is given by the excitation beam. The high sensitivity and minimal photobleaching of light sheet fluorescence microscopy are ideally suited for live-cell imaging experiments. Furthermore, application of this technique would open possibilities for a more detailed investigation of HIV-1 cell‑to-cell transmission in 3d tissue cultures. In *in vivo* systems, directed virus release and transmission through direct contacts between infected cell and uninfected cells (so-called virological synapses) represents an effective pathway of HIV-1 transmission (for reviews see [101,102,103,104]). However, quantitative kinetic analyses of HIV-1 budding site formation have so far been limited to TIRF microscopy, which cannot visualize intracellular synapses, and were restricted to flat adherent model cell lines instead of the physiological target cells. Light sheet microscopy may provide a means for detailed analysis of HIV-1 particle formation and transmission between cells of the immune system, or even between cells in organotypic histocultures. With continuous improvements in the properties and functionalities of labels for live-cell imaging and advances in fluorescence microscopy and image analysis methods, the future for quantitative live-cell imaging in HIV-1 research looks very bright.
